# Adolescent Refugee Potential Traumatic Experience and Mental Health in Gambella Region in Ethiopia: A Model Examining Mediating Effects of Coping and Resilience

**DOI:** 10.3390/healthcare13091069

**Published:** 2025-05-06

**Authors:** Solomon D. Danga, Babatope O. Adebiyi, Erica Koegler, Conran Joseph, Nicolette V. Roman

**Affiliations:** 1 Centre for Interdisciplinary Studies of Children, Families and Society, University of the Western Cape, Private Bag x17, Bellville 7535, South Africa; 3571010@myuwc.ac.za (B.O.A.); nroman@uwc.ac.za (N.V.R.); 2 School of Social Work, University of Missouri St. Louis, 1 University Blvd, St. Louis, MO 63121-4400, USA; koeglere@umsl.edu; 3 Division of Physiotherapy, Faculty of Medicine and Health Sciences, Stellenbosch University, Cape Town 7602, South Africa; conran@sun.ac.za

**Keywords:** trauma, coping, resilience, mental health, refugee, adolescent, depression, anxiety, stress, mediation, moderation

## Abstract

**Background**: Refugees often experience multiple traumatic events due to persecution, conflict, and displacement, which can result in poor mental health outcomes. **Objective:** The current study examined whether coping and resilience mediate the relationship between traumatic experience(s) and mental health outcomes and whether these indirect effects were moderated by age, gender, and refugee camp duration. **Method:** A cross-sectional, correlational study design was employed. Data were collected from 14 July 2019 to 28 August 2019. A sample of 414 adolescent refugees from two refugee camps in the Gambella regional state of Ethiopia were selected using proportional stratified sampling and simple random sampling techniques. Participants completed a self-reported questionnaire. Data were analysed using Structural Equation Modelling for hypotheses testing causal models. **Results:** Coping and resilience fully mediated the relationship between traumatic experience and mental health among adolescent refugees. Refugee camp duration as a moderator factor weakens the positive relationship between traumatic experiences and mental health outcomes. **Conclusions**: This study highlights the critical importance of comprehensive intervention strategies that strengthen adolescent refugee personal, family, social, and community level coping and resilience abilities within refugee camps setting. The findings also strongly suggested that early intervention in refugee camps could protect adolescent refugees from possible psychological distress and maintain adolescents’ mental health and well-being within refugee camps.

## 1. Introduction

United Nations Higher Commissioner for Refugees [1] figure in sub-Sahara regions estimated that the proportion of children and adolescents among the refugees hosted in these regions is above 50%. Refugees often experience multiple traumatic events due to persecution, conflict, and displacement, which can result in poor mental health outcomes [2,3]. Ethiopia hosts Africa’s third-largest number of refugees [4]. These refugee groups live in highly concentrated refugee camps with insufficient mental health care and protection. Few studies were conducted in the Ethiopian context about refugee mental health problems prevalence rate, caregiver mental health, and risk and protective factors, particularly among adolescents exposed to different traumatic events [5,6]. Thus, far too little attention has been paid to the mental health of children and adolescent refugees hosted in Africa. Studies also showed individuals who develop coping strategies and resilience may buffer some of the impacts of traumatic experiences [7,8,9]. There is a dearth of research on the mediating effects of coping and resilience on the relationship between traumatic experience and poor mental health among adolescent refugees in the African refugee camps context. Therefore, this study has two objectives: (i) to examine the mediating effects of coping and resilience and (ii) to determine the moderating effects of age, gender, and refugee duration on the association between trauma and mental health outcomes in a sample of adolescents in refugee camps in Gambella Regional state in Ethiopia to inform future interventions with adolescent refugees.

### 1.1. Potential Traumatic Experience in Adolescent Refugee

Traumatic events pose a serious and major threat to a person’s life. This includes exposure to actual or threatened death, a serious injury, losses in violent circumstances, and/or witnessing such events [10]. Traumatic events include war, torture, rape, violent assault, forced labour, and serious accidents [11,12]. There is no clear-cut demarcation between traumatic events and other adversities among refugee survivors who have usually experienced multiple traumatic events and adversities [10]. Refugees resettling in a new country are among the most vulnerable groups in society. Because of resettlement in a host country, refugees face new challenges related to school integration, language, and cultural adjustment [13,14,15]. Adolescent refugees may be particularly vulnerable to adverse physical and mental health issues than adult refugees due to being in an unstable stage of development characterised by major changes in physical, cognitive, and psychosocial development changes and trauma experienced, witnessed, and heard about in their migration [16].

Children and adolescent refugees migrate with histories of exposure to trauma. Such trauma may include the violent death of a parent, injury to or torture of a family member, separation from parents, the disappearance of loved ones, enduring political oppression, deprivation of human rights and education, witnessing murder or massacre, exposure to bombardments, terrorist attack, forcible eviction from home, and detention [17]. Young refugees may also experience physical injury and disability inflicted by violence, sexual assault, and subjection to child-soldier activities. As a result, many health risk factors may arise and manifest [2]. Studies also found that both traumatic events and daily stressors contribute substantially to psychopathologies in refugees and asylum-seekers, including post-traumatic stress disorder (PTSD), depression, anxiety, and somatization [3,5,15].

### 1.2. Relationship Between Potential Traumatic Experience and Mental Health

Studies on the association between traumatic experience and poor mental health outcomes found a positive relationship between these variables among the refugee population. For instance, studies conducted in Norway and South Korea found a positive association between different traumatic experiences and depression and anxiety among adolescent refugees [18,19]. Another study conducted in the United States also found a significant positive association between exposure to war trauma and depressive mood in adolescent refugees [20]. However, there may be unknown mediating and moderating factors in this relationship. Identifying these factors could help to develop an intervention strategy for better mental health outcomes and adjustment in the new country.

### 1.3. Mediating Effects of Coping and Resilience in Adolescent Refugee Mental Health

There is increasing interest in investigating mediators of traumatic experience effects on mental health. For instance, the mediating effects of coping [19] and resilience [21,22] were examined among refugee youth populations. Studies examining the mediation effects of coping strategies on mental health outcomes were contradictory. For example, a study conducted in Turkey on adolescent and adult Syrian refugees found that none of the coping strategies significantly mediated the relationship between traumatic experiences and psychological distress [21]. However, another study conducted in Ethiopia’s Mai Aini refugee camp found that pre-migration and post-migration traumatic events were indirectly associated with PTSD symptoms through task-oriented coping styles [23].

Similarly, studies on the mediation effect of resilience on the association between traumatic experience and mental health were also contradictory. A study conducted on North Korean youth refugees in South Korea found that the indirect effect of ego resiliency between traumatic event exposure and mental health was not statistically significant [19]. In contrast, a study comparing refugees in Germany and German residents found that lower resilience partially accounts for group differences of higher psychological distress in refugees [24].

### 1.4. Moderating Effects of Age, Gender, and Refugee Camp Duration

There is not enough literature to clarify the moderate effects of age, gender, and refugee camp duration on the relationship between traumatic experience and mental health among adolescent refugees. Therefore, the independent effects of these moderator variables on the mental health of young refugees were discussed as follows: studies on young refugees’ age as a possible contributing factor to mental health outcomes were inconclusive. Three studies found that older youth had worse mental health outcomes than younger youth [25,26,27]. However, one study found that 9- to 10-year-old children had the highest depressive symptoms compared to the adolescent group [28]. Two studies found that age did not affect mental health outcomes [29,30].

Concerning gender differences, two studies reported a higher rate of psychopathology in girls than in boys in relation to emotional problems such as anxiety and depression [31,32]. Another study also found that symptoms of post-traumatic stress, anxiety, and depression showed a greater decrease in boys relative to girls over time [30]. However, most studies conducted among young refugees found no significant difference between boys and girls on PTSD [33], PTSD and depression [34], and depression and anxiety [35].

A systematic review conducted on young refugees reported that no consistent pattern of mental health symptomatology was seen across variations in the duration of refugee camp living [3]. For instance, one study found that the length of time living in camps was associated with more severe PTSD symptoms [36]. Another study also showed that the longer one stays in a refugee camp, the more severe the psychological problems or post-traumatic stress symptoms [25]. In contrast, a study on war-related stress affected refugee children in Croatia and reported that youth who lived in camps longer had fewer PTSD symptoms than those who lived in camps for a shorter period. These authors also suggest that a protracted duration in refugee camps without adequate safety, support, and services could potentially maintain or exacerbate PTSD and other trauma symptoms in children over time [26].

### 1.5. The Current Study

The overall aim of the current study was (i) to examine the mediating effects of coping and resilience, and (ii) to determine the moderating effects of age, gender, and refugee duration on the association between trauma and mental health outcomes framed within Moos’s context, coping, and adaptation model. This model provides a theoretical framework for exploring contextual and social factors and relationships associated with adolescent health and well-being [37]. Moos’s model of context, coping, and adaptation consists of five panels that determine the health and well-being of adolescents. Panel I (environmental system) is composed of the social climate, which includes both daily life stressors (such as work stress) and social resources (such as family members, friends, groups, and organisations). Panel II (personal system) involves socio-demographic characteristics and personal coping resources (such as cognition, self-confidence, social beliefs, and other traits-related dimensions). Panel III (transitory conditions) includes the combined influences of environmental and personal factors (such as life events and intervention programs). Panel IV (cognitive appraisal and coping skills) is shaped by the relationships between environmental, personal, and transitory conditions. Panel V (health and well-being) is the outcome of the model’s inter-relationship among all four panels. Moos’s model also indicates bidirectional relationships among the panels [37,38].

**Figure 1:**
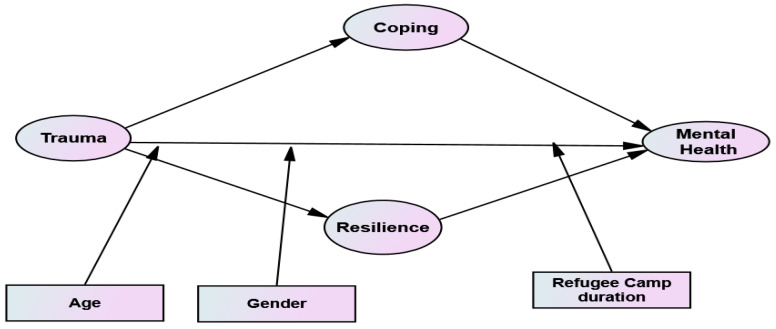
Conceptual Model.

In light of Moos’s determinants of the health and well-being of adolescents, as it is shown in Figure 1, this study intends to use panel III (transitory conditions), panel IV (cognitive appraisal and coping skills), and panel V (health and well-being). The combined influences of panel I and panel II resulted in panel III so that for model testing, panel I and II will not be directly used to eliminate the repetition of concepts. Panel III (transitory conditions) is related to the degree of traumatic life experienced by adolescents. Panel IV (cognitive appraisal and coping skills) in the context of the present study is related to coping strategies used, and the resilience ability adolescents develop after exposure to trauma. Panel V (health and well-being) is intended to see adolescents’ mental health outcomes (such as stress, anxiety, and depression). Hence, based on Moos’ model as a theoretical framework and guided by the literature reviews, the following hypotheses were formulated:

**H1.**  *Coping mediates the positive relationship between traumatic experience and mental health.*

**H2.**  *Resilience mediates the positive relationship between traumatic experience and mental health.*

**H3.**  *Exposure to traumatic experience has a positive effect on mental health.*

**H4.**  *Age moderates the relationship between traumatic experiences and mental health.*

**H5.**  *Refugee camps duration has a moderating effect on the relationship between traumatic experience and mental health.*

**H6.**  *Gender moderates the relationship between traumatic experience and mental health.*

Young refugees’ traumatic experiences and poor mental health outcomes have become a growing health concern in humanitarian settings [3]. The present study will assist in identifying mediating and moderating factors in the relationship between trauma and mental health of youth refugees framed within Moos’s model of context, coping, and adaptation. In addition, this study will contribute to the existing knowledge in identifying and implementing interventions that improve youth coping and resilience factors that may reduce the risk of psychopathology following traumatic experiences.

## 2. Materials and Methods

### 2.1. Participants

A cross-sectional, correlational design was used to test mental health models based on potential traumatic experience, coping, and resilience of adolescent refugees in refugee camps in Ethiopia. There was a total of eight refugee camps in the Gambella regional state in Ethiopia during data collection. These refugee camps were insecure, with frequent conflict within the camps as well as within the local hosting community. Two refugee camps were selected using convenience sampling for safety and security reasons as advised by local experts and the large number of adolescent refugees hosted in the camp.

A proportional stratified random sampling technique was employed to select representative samples of adolescent refugees aged 12-18 based on the refugee camps which formed part of the sampling frame. Fifteen percent of the samples for the primary study were selected for the pilot study to test the reliability of the instruments. The questionnaire was administrated in English language since the South Sudanese refugees hosted in Ethiopia used English as a native speaker. After the completion of the questionnaire for the pilot study, interviews were conducted on 10 participants to identify language barriers, difficult items, challenging parts of the questionnaire, length of the items/questionnaire, and the appropriateness of the time allotted to fill the questionnaire. Adolescent refugees within two refugee camps were then stratified according to age and gender. The sample was drawn proportionally from the identified stratum of age and gender using a simple random sampling technique. The final sample comprised 414 adolescent refugees; 235 (56.8%) males and 179 (43.2%) females participated in the study.

### 2.2. Measures

#### 2.2.1. Demographic Characteristics

Demographic details regarding the participant’s age, gender, and length of refugee camp duration in years were collected in this study.

#### 2.2.2. Harvard Trauma Questionnaire (HTQ)

Harvard trauma questionnaire part I [39] comprises 17 types of maltreatment and war events, rated based on proximity to the events (experienced = 3, witnessed = 2, heard about = 1, and no involvement = 0). Higher scores for each item indicate a higher experience of trauma. The refugee’s traumatic events fell into four general factors based on a study of Southeast Asian refugees. These factors are (i) material deprivation (three events: lack of food or water, lack of shelter, and ill health without access to medical care); (ii) violence to others (four events: unnatural death of family member or friend, murder of family member or friend, murder of stranger, combat situation); (iii) physical/bodily injury (four events: torture, serious injury, rape or sexual assault, other types of humiliation); and (iv) coercion (six events: imprisonment, brainwashing, lost or kidnapped, being close to death, forced isolation, forced separation from family members) [39]. Example items are item 1 “Lack of food or water” and item 11 “Forced separation from family members”. In this study, composite factor scores were used for each factor. The Harvard Trauma questionnaire is a cross-culturally validated instrument to measure torture and trauma in the refugee context. In the present study, we computed the internal reliability of the items of scale using Cronbach’s alpha and found reliability values of 0.82, 0.84, 0.80, 0.70, and 0.85 for the subscale material deprivation, violence to others, physical/bodily injury, coercion, and the overall scale, respectively.

#### 2.2.3. Brief COPE

The Brief COPE [40] scale is a self-report short version questionnaire. The scale has 14 subscales categories into three coping styles. These are (i) problem-focused strategies (includes active coping, instrumental support, planning, and positive-reframing); (ii) emotion-focused strategies (including acceptance, emotional support, self-blame, humour, venting and religion); and (iii) avoidant or dysfunctional strategies (includes self-distraction, behavioural disengagement, denial, and substance use). Response options range from 0 (I have not been doing this at all) to 3 (I have been doing this a lot). Sample items from Brief COPE scales are item 5 “I got emotional support from others”, item 18 “I accepted the reality of the fact that it has happened”, and item 28 “I criticized myself”. Brief COPE was administered in a dispositional response format to assess coping style in the present study. Participants were asked to recall how they usually responded to traumatic and stressful situations in their migration experience. A composite factor scores were generated for each subscale. In the present study, internal consistency of the subscales was computed; Cronbach’s alpha of the subscales was 0.70, 0.75, and 0.68 for problem-focused, emotion-focused, and dysfunctional coping strategies, respectively.

#### 2.2.4. Connor–Davidson Resilience Scale (CD-RISC-10)

Connor–Davidson Resilience Scale (CD-RISC-10) [41] is a unidimensional self-report measure that consists of 10 items. CD-RISC-10 measures the ability to bounce back from stressful events or traumatic life experiences. Items were rated on a 5-point Likert scale from 0 (not true at all) to 4 (true nearly all the time), with higher scores reflecting increased resilience ability. Example items are “Deal with whatever comes my way’’ and “Able to handle unpleasant feelings”. A composite factor score was generated, and the Cronbach’s alpha value of the scale was 0.83 in the present study.

#### 2.2.5. Depression Anxiety Stress Scales (DASS-21)

The Depression Anxiety Stress Scales (DASS-21) is a 21-item questionnaire, including three self-report scales designed to measure the negative emotional states of depression, anxiety, and stress as indicators of mental health construct. Each of the three sub-scales contains seven items that respondents were asked to use 4-point severity scales to rate the extent to which they have experienced each state from 0 (did not apply to me) to 3 (applies to me very much) [42]. Example items from stress, depression, and anxiety sub-scale items, respectively, are “I found it difficult to relax”, “I felt that life was meaningless”, and “I was aware of dryness of my mouth”. In this study composite factor scores were used for each factor. In the present study, Cronbach’s alpha values of the subscales were 0.67, 0.77, and 0.74 for depression, anxiety, and stress, respectively.

### 2.3. Procedure

Ethical approval was obtained from the Humanities and Social Science Research Ethics Committee (ethical clearance: HS19/5/12) of the University of the Western Cape. The Agency for Refugees and Returnees Affairs-Ethiopia (ARRA) granted permission to conduct the study in the refugee camps in Ethiopia. The camp directors and protection officers then granted permission to conduct the study at their respective refugee camps. Data were collected from 14 July 2019 to 28 August 2019 by the principal investigator and assistant data collectors. The consent form was completed and signed by parents/caretakers or guardians before administering the questionnaire to adolescents who volunteered to participate in the study. Adolescent refugees were invited to sign an assent form to participate in this study. Participants were informed not to disclose their identity during the data collection process by not writing their names to assure anonymity and confidentiality of the information. Furthermore, participants were given the right to withdraw at any time of the research process without prejudice or negative consequences. There was no compensation paid to participants in this study.

### 2.4. Data Analysis

The collected data were entered, cleaned, and analysed using Statistical Package for Social Science IBM SPSS Statistics version 26.0. Assumptions of normality, linearity, and multicollinearity were also checked. Internal reliability of items was computed using Cronbach alpha to evaluate the reliability of the scales and subscales of the measuring instruments. Correlations between the constructs of this study were also calculated to assess the discriminant and convergent validity. Composite factor scores were generated by imputing factors for all the models tested in this study. The dataset was then imported into Analysis of Moment Structures IBM SPSS AMOS version 26 software where a preliminary confirmatory factor analysis was conducted to evaluate the fitness of the measurement model of the latent constructs, followed by Structural Equation Modelling to test the proposed hypotheses.

## 3. Results

### 3.1. Confirmatory Factor Analysis

The researchers run a preliminary confirmatory factor analysis (CFA) for the proposed model’s latent constructs using the Analysis of Moment Structures (AMOS) version 26. The Confirmatory Factor Analysis was conducted to evaluate the fitness of the latent construct’s measurement model. The convergent and discriminant validity was assessed by conducting a correlation matrix. In Table 1, the correlation matrix suggests that traumatic experience sub-scales, coping strategies sub-scales, and resilience and mental health sub-scales were less than 0.85 indicating the convergent and divergent validity of the variables entered in the model. CFA procedure produces several measurement model assessment measures using the data collected, and in the proposed theoretical model, a Chi-square value (*X*^2^*/df*) = 1.55 was obtained. The value of the Chi-square indicated that the data fit the measurement model. The goodness-of-fit indices also indicated a good model fit: Comparative Fit Index (*CFI*) = 0.91, Standardised Root Mean Residual (*SRMR*) = 0.06, and Root Mean Square Error of Approximation (*RMSEA*) = 0.03 [43,44].

### 3.2. Hypotheses Testing: Structural Equation Model

#### 3.2.1. The Mediating Effects of Coping and Resilience

**Figure 2:**
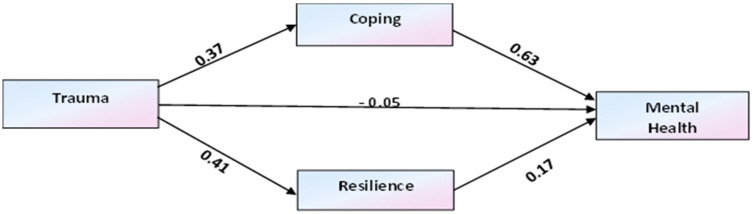
Mediation effects of coping and resilience.

In this study, the first model examines the mediating effects of coping and resilience on the associations between traumatic experience and mental health. The structural equation model results are presented in Table 2 and Figure 2. The structural model goodness of fit indices indicated an acceptable fit value (Chi-square value (*X*^2^/*df)* = 4426 Comparative Fit Index (*CFI*) = 0.991, Standardised Root Mean Residual (*SRMR*) = 0.042, Root Mean Square Error of Approximation (*RMSEA*) = 0.091). The proposed hypotheses were tested and analysed based on the regression path coefficient and *p*-value of the causal relationships in the model. The analysis indicated that hypotheses (H1 and H2) were supported. Mediating variables coping (*β* = 0.23, *p* < 0.01) and resilience (*β* = 0.06, *p* < 0.001) have a significant mediating effect on the association between traumatic experience and mental health. On the other hand, the hypothesis for the direct effect of traumatic experience on mental health was not significant (*β* = −0.05, *p* = 0.207). These results indicated that coping and resilience fully mediate the relationship between traumatic experience and mental health (see Table 2).

The total effects of traumatic experiences, coping, and resilience on mental health are equal to the sum of all variables’ indirect and direct effect. Table 3 depicted the direct, total effects, and total indirect effects of trauma, coping, and resilience on mental health. Based on the unstandardised regression weight estimates, the total effect of trauma, coping, and resilience was significant (*β* = 0.25, *p* < 0.001) and the total indirect effect of coping and resilience on mental health was also significant (*β* = 0.29, *p* < 0.001). Hence, all the direct and indirect effects contribute to the mental health of adolescent refugees.

#### 3.2.2. Moderation Effect of Age and Refugee Camps Duration

In this moderating model, Structural Equation Modelling (SEM) analysis was adopted. Age and refugee camp duration are moderator variables, and traumatic experience and mental health are independent and dependent variables. Besides the predictor variable, the researchers need to create an interaction variable which is the product of the predictor and moderator variable. As a result, two interaction variables were computed from the products of age, refugee camp duration, and traumatic experiences.

**Figure 3:**
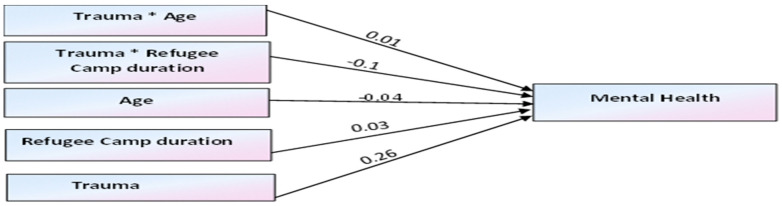
Moderation effects of age and refugee camp duration on mental health.

The results of the structural equation model are presented in Table 4 and Figure 3. The structural model goodness of fit indices indicated an acceptable fit value (Chi-square value (*X*^2^/*df*) = 4643, Comparative Fit Index (*CFI*) = 1.00, Standardised Root Mean Residual (*SRMR*) = 0.00, and Root Mean Square Error of Approximation (*RMSEA*) = 0.09). The proposed hypotheses were tested and analysed based on the regression path coefficient and *p*-value of the causal relationships in the model. The analysis indicated that the hypothesis for the moderation effect of age on the relationship between traumatic experience and mental health was not significant (*β* = 0.01, *p* = 0.83). However, there was a significant main effect between trauma and mental health (*β* = 0.26, *p* < *0*.001), and the moderating effect of refugee camp duration was also significant (*β* = −0.10, *p* < 0.05). In this model, partial moderation occurred since the hypothesis for the direct effect of traumatic experience on mental health was still significant after the moderators entered the model.

**Figure 4:**
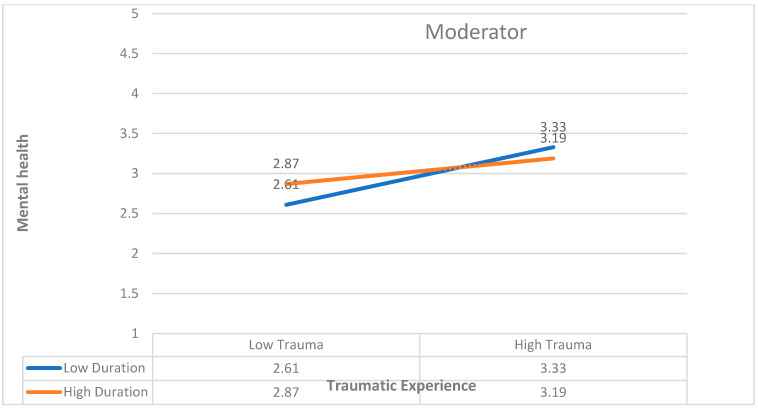
A visual display of the interaction between traumatic experience and refugee camps duration on mental health.

Further two-way effect analysis was conducted for the significant effect of refugee duration on the relationship between traumatic experience and mental health to determine the magnitude and direction of the moderating effect using Stats Tools Package [45] as shown in graph Figure 4. The result indicated that refugee camps duration dampens the positive relationship between traumatic experience and the mental health of adolescents. So, for those with shorter refugee camp duration, trauma had a positive effect on poor mental health, and for those with longer refugee camp duration, the effect of trauma on mental health was less.

#### 3.2.3. Moderation Effect of Gender

Multi-group structural equation modelling has been applied to examine the moderate effect of gender in this model. In multi-group SEM, moderators are examined by dividing the sample into sub-samples (male and female in this case). The moderating effect of gender differences was examined by the multi-group analysis of path coefficient difference using Multi-group Analysis AMOS Plugin [46]. The structural model goodness of fit indices indicated good fit values (Chi-square value (*X*^2^/*df*) = 2357, Comparative Fit Index (*CFI*) = 0.99, Standardised Root Mean Residual (*SRMR*) = 0.04, and Root Mean Square Error of Approximation (*RMSEA*) = 0.05). The global Chi-square test of difference was conducted at the model level, and the result indicated that the *p*-value of the chi-square difference test was not significant. This result implied that groups are not different at the model level; however, they may be different at the path level. When the path level moderation effect of gender was examined, the result also showed that there is no significant difference between males and females on the positive relationship traumatic experience and mental health. Thus, H6 is not supported.

## 4. Discussion

The present study examined whether traumatic experience has a unique effect on mental health or whether mental health will be mediated by coping and resilience with respect to traumatic experiences. Besides, it is also tested whether the indirect effects linking traumatic experience and mental health through coping and resilience will be moderated by age, gender, and refugee camps duration.

### 4.1. The Mediating Effects of Coping and Resilience

The findings of this study indicated that coping and resilience mediates the relationship between traumatic experience and mental health. This result implied that coping and resilience buffered traumatic experiences of adolescent refugees from possible poor mental health outcomes in this study. Regarding the mediation effect of coping, this result was consistent with the study conducted in Mai Aini refugee camp in Ethiopia, which found that pre-migration and post-migration traumatic events were indirectly associated with PTSD symptoms through task-oriented coping styles [23]. However, it contradicted a study conducted in Turkey on adolescent and adult Syrian refugees, which reported that none of the coping strategies significantly mediated the relationship between traumatic experiences and psychological distress [21].

With regards to the mediation effect of resilience, a comparative study conducted between refugees in Germany and German residents found that lower resilience partially accounts for group differences of higher psychological distress in refugees [24]. The current study indicates that resilience mediates the relationship between traumatic experiences and mental health. However, this result does not support research on North Korean youth refugees in South Korea that found the indirect effect of ego resiliency between trauma exposure and mental health was not statistically significant [19]. This could be due to the fact that this study was based on only ego resiliency instead of taking into account the social and cultural factors of resilience [19].

Adolescent refugee participants in this study develop coping and resilience despite the traumatic experience exposed. These may be attributed to adolescent refugee’s strong parental and social support as well as individual and community resources. For instance, Windle [47] reported individual factors (such as temperaments, self-concept, emotions, and social skills), family factors (such as attachment, communication, parental relations, and parenting style) and social environment factors (such as social conditions, inclusion, access, and involvement) determine youth resilience. Thus, intervention strategies employed in participants of this study should focus on these determinants of resilience factors.

### 4.2. Moderation Effects of Age, Gender, and Refugee Camps Duration

This study shows that refugee camp duration reduces the positive relationship between traumatic experiences and adolescents’ mental health. However, the interaction effect of age and gender on the relationship between trauma and mental health was not significant. This result of refugee camp duration supports Ajdukovic and Ajdukovic [26] findings which indicated that youth who lived in camps longer had fewer PTSD symptoms than those who lived in camps for a shorter time. On the contrary, another research result showed that the longer one stays in a refugee camp, the more severe the psychological problems or post-traumatic stress symptoms [25]. Thus, adolescent participants of this study hosted in the refugee camps need early intervention to reduce the positive impact of traumatic experiences on poor mental health. In addition, early intervention strategies should focus on better adjustment and integration with the local host community, which weakens the positive effect of traumatic experiences on poor mental health outcomes.

The result of this study, where age was found to be a moderator variable, confirms the findings of previous studies, which indicated that age did not have an effect on mental health outcomes [29,30]. However, it contradicted the findings of previous studies, which indicated that older youth had worse mental health outcomes than younger [25,26,27]. It also does not support the finding that younger youths had the highest depressive symptoms compared to older adolescents [28]. Similarly, the findings on gender were also consistent with the results, which indicated no significant difference between boys and girls on PTSD [33], PTSD and depression [34], and depression and anxiety [35]. However, it contradicts the results of two studies that reported a higher rate of psychopathology was found in girls than in boys [31,32]. It is also contradicted with [30] which found that symptoms of post-traumatic stress, anxiety, and depression showed a greater decrease in boys relative to girls over time. The findings of this study implied that demographic variables of age and gender did not have moderation effects on the association between traumatic experience and mental health outcomes. This finding may be explained by similarities in traumatic events experienced by participants of the study and resettlement situations in the host refugee camps. Thus, age and gender did not moderate the association between traumatic exposure and poor mental health outcome in this study.

## 5. Strengths and Limitations

The study had several strengths. First, the results were based on representative samples drawn randomly in refugee camps. Second, it is one of the first few studies conducted on young refugees mediating and moderating factors on the relationship between traumatic experience and mental health outcomes in refugee camps. The study also has some limitations. First, the cross-sectional nature of the study was its main limitation. It cannot provide evidence of causal relationships but relationships among the study variables. It is crucial to explore the potential causal relationships and mechanisms behind these effects on young adolescent refugees’ traumatic experiences, coping, resilience, and mental health. Second, the results were based on self-reported data, which may be associated with recall bias. This could be attributed to the nature of self-reported measurements that participants had imperfect memory of past events/experiences, or other personal biases. Further studies should be based on more objective measures of both exposures and outcomes to verify the hypotheses in the present study. Finally, participants of this study were representative samples of South Sudanese adolescent refugees hosted in selected refugee camps in Ethiopia. Thus, the findings of this study may not be generalisable to other refugee camps.

## 6. Recommendations for Future Studies

This study addressed the mediation effects of coping and resilience and moderation effects of demographic factors of age, gender, and refugee duration on the association between traumatic experience and poor mental health among adolescent refugees in Gambella region in Ethiopia. The findings of this study could provide insight on the mediating and moderating factors of mental health outcomes among adolescent refugees for mental health professionals to improve the service delivered to adolescent refugees. Furthermore, it may help policymakers to design and implement proper preventive and intervention strategies to enhance adolescents’ mental health and well-being in refugee camps. For instance, developing specific intervention guidelines for refugee adolescents exposed to traumatic experiences, delivering training and developing mental health professionals’ skills on trauma-focused intervention strategies, and creating trauma-focused intervention centres within the refugee camps. It is recommended that future research should consider other risk factors such as social isolation, loneliness, parental separation and protective factors such as positive family functioning, social support, community support, and physical activities on the association between adversities and poor mental health among adolescent refugees to have a wider picture of this research area in the African context.

## 7. Conclusions

This study shows that coping and resilience fully mediated the relationship between trauma and coping among young refugees sheltered in refugee camps. Thus, this study underscores the critical importance of comprehensive intervention strategies that strengthen adolescent refugee personal, family, social, and community level coping and resilience abilities within refugee camp settings. In addition, the results of this study provide evidence for the need for early intervention in refugee camps that can protect adolescent refugees from potential psychological distress and promote their mental health and well-being within refugee camps.

## Figures and Tables

**Table 1:** Constructs correlation matrix.

Variable	1	2	3	4	5	6	7	8	9	10	11
1	-										
2	0.27 ***	-									
3	0.27 ***	0.24 ***	-								
4	0.37 ***	0.42 ***	0.45 ***	-							
5	0.04	0.21 ***	−0.01	0.09	-						
6	0.28 ***	0.27 ***	0.11 *	0.16 ***	0.49 ***	-					
7	0.26 ***	0.17 ***	0.10 *	0.13 **	0.51 ***	0.71 ***	-				
8	0.11 *	0.31 ***	0.03	0.12 **	0.34 ***	0.32 ***	0.21 ***	-			
9	0.16 ***	0.19 ***	0.01	0.09	0.22 ***	0.39 ***	0.30 ***	0.50 ***	-		
10	0.08	0.04	0.04	0.03	0.14 **	0.21 ***	0.24 ***	0.42 ***	0.42 ***	-	
11	0.04	0.03	−0.07	0.01	0.10*	0.05	0.03	0.22 ***	0.13 **	0.11 *	-

*** *p* < 0.001, ** *p* < 0.01, * *p* < 0.05. 1 = Material deprivation, 2 = violence to others, 3 = physical/bodily injury, 4 = coercion, 5 = problem-focused coping, 6 = emotion-focused coping, 7 = dysfunctional coping, 8 = resilience, 9 = stress, 10 = anxiety, 11 = depression.

**Table 2:** Hypothesis testing for mediation model.

Hypotheses	Beta	Std. Error	*p*-Value	95 % CI	
				Lower	Upper
Trauma→Coping→Mental health	0.23 **	0.03	0.010	0.18	0.32
Trauma→Resilience→Mental health	0.06 ***	0.02	0.001	0.03	0.11
Trauma→Mental health	−0.05	0.04	0.279	−0.14	0.004

** *p* < 0.01, *** *p* < 0.001. CI: confidence interval.

**Table 3:** Summary table of the direct, indirect, total effect, and total indirect effect.

Model	Beta	Std. Error	*p*-Value	95 % CI	
				Lower	Upper
**Direct effects**					
Trauma→Coping	0.37 ***	0.02	0.001	0.14	0.23
Trauma →Resilience	0.41 ***	0.07	0.000	0.53	0.28
Trauma→Mental health	0.29 ***	0.04	0.001	0.17	0.38
Coping→Mental health	0.63 ***	0.08	0.001	1.23	1.56
Resilience→Mental health	0.17 ***	0.02	0.001	0.13	0.25
**Total direct effect**					
Trauma→Mental health	0.25 ***	0.05	0.001	0.17	0.38
**Total indirect effect**					
Trauma→Mental health	0.29 ***	0.04	0.001	0.25	0.41

*** *p* < 0.001.

**Table 4:** Hypothesis testing for age refugee camp duration as moderators.

Hypotheses	Beta	Std. Error	*p* Value	95% CI	
				Lower	Upper
Trauma_X_Age→Mental health	0.01	0.03	0.80	−0.06	0.07
Trauma_X_Refugee camp duration→Mental health	−0.10 *	0.04	0.03	−0.16	−0.02
Age→Mental health	−0.04	0.06	0.39	−0.14	0.09
Refugee camp duration→Mental health	0.03	0.06	0.61	−0.08	0.14
Trauma→Mental health	0.26 ***	0.05	0.001	0.16	0.35

* *p* < 0.05, *** *p* < 0.001.

## Data Availability

All data generated and analysed are included in the manuscript.
